# Antibody Production in Response to Staphylococcal MS-1 Phage Cocktail in Patients Undergoing Phage Therapy

**DOI:** 10.3389/fmicb.2016.01681

**Published:** 2016-10-24

**Authors:** Maciej Żaczek, Marzanna Łusiak-Szelachowska, Ewa Jończyk-Matysiak, Beata Weber-Dąbrowska, Ryszard Międzybrodzki, Barbara Owczarek, Agnieszka Kopciuch, Wojciech Fortuna, Paweł Rogóż, Andrzej Górski

**Affiliations:** ^1^Bacteriophage Laboratory, Ludwik Hirszfeld Institute of Immunology and Experimental Therapy, Polish Academy of SciencesWrocław, Poland; ^2^Phage Therapy Unit, Medical Center of the Ludwik Hirszfeld Institute of Immunology and Experimental Therapy, Polish Academy of SciencesWrocław, Poland; ^3^Department of Clinical Immunology, Transplantation Institute, Medical University of WarsawWarsaw, Poland

**Keywords:** antibodies, antimicrobials, ELISA, immune response, phage cocktail, phage therapy, Staphylococcal Infections

## Abstract

In this study, we investigated the humoral immune response (through the release of IgG, IgA, and IgM antiphage antibodies) to a staphylococcal phage cocktail in patients undergoing experimental phage therapy at the Phage Therapy Unit, Medical Center of the Ludwik Hirszfeld Institute of Immunology and Experimental Therapy in Wrocław, Poland. We also evaluated whether occurring antiphage antibodies had neutralizing properties toward applied phages (K rate). Among 20 examined patients receiving the MS-1 phage cocktail orally and/or locally, the majority did not show a noticeably higher level of antiphage antibodies in their sera during phage administration. Even in those individual cases with an increased immune response, mostly by induction of IgG and IgM, the presence of antiphage antibodies did not translate into unsatisfactory clinical results of phage therapy. On the other hand, a negative outcome of the treatment occurred in some patients who showed relatively weak production of antiphage antibodies before and during treatment. This may imply that possible induction of antiphage antibodies is not an obstacle to the implementation of phage therapy and support our assumption that the outcome of the phage treatment does not primarily depend on the appearance of antiphage antibodies in sera of patients during therapy. These conclusions are in line with our previous findings. The confirmation of this thesis is of great interest as regards the efficacy of phage therapy in humans.

## Introduction

Phage treatment is considered one of the most promising therapies in fighting human pathogenic bacterial strains, including those that are antibiotic resistant such as methicillin-resistant *Staphylococcus aureus* (MRSA). Due to constantly declining effectiveness of antibiotics, pathogenic bacteria have become endemic organisms, especially in medical care units (Arnold et al., 2011; Cheon et al., 2016). Regardless of such worldwide increasing microbiological hazard, the wider use of phages faces skepticism over its effectiveness, as it was postulated that human antibodies may have a negative effect on favorable treatment results (Sulakvelidze et al., 2001). Herein, we estimate the induction of antiphage antibodies and their potential neutralizing influence on *S. aureus* MS-1 phage cocktail.

Data regarding the antiphage humoral response during phage treatment are still extremely scarce. Only a few articles describe antibacteriophage activity of human sera of patients during phage treatment and healthy volunteers (Kucharewicz-Krukowska and Ślopek, 1987; Bruttin and Brussow, 2005; Górski et al., 2007; Łusiak-Szelachowska et al., 2014). Kucharewicz-Krukowska and Ślopek (1987) reported that induction of antiphage antibodies was detected in 54.4% of patients during therapy (the 10th day of phage treatment, oral administration). Only in 3 of 57 patients (5.3%) did sera indicate high antiphage activity. Similarly, in the experiment by Bruttin and Brussow (2005), none of the examined volunteers showed an increased level of antiphage antibodies (IgG, IgA, and IgM) after oral administration. Among 122 patients from the Phage Therapy Unit in Wrocław, only 15 of them (12.3%) demonstrated high (*K* > 18) antiphage activity of sera (AAS), mostly during local administration (Łusiak-Szelachowska et al., 2014). The same report showed no clear correlation between phage administration and increased level of antiphage antibodies in patients’ sera evaluated by absorbance measurements using the ELISA test. Further studies (Łusiak-Szelachowska et al., 2016) clearly showed that AAS depends on the route of phage administration. All patients receiving staphylococcal phage preparations orally showed a low level of AAS, whilst those with local administration of phage cocktail had high AAS in almost half of the cases. Generally, use of a phage cocktail resulted in a stronger immune response than monotherapy. With regard to the relatively small number of patients with such high activity of sera, it is difficult to define the relationship between serum antiphage activity, the level of antiphage antibodies and effectiveness of the phage therapy. It has to be said that induction of antiphage antibodies and their binding to phage antigens does not necessarily mean the loss of phage viability (Górski et al., 2012).

Our group (Łusiak-Szelachowska et al., 2014) published probably the first report where the production of antiphage antibodies was compared to their neutralizing properties and was related to the clinical outcome in patients receiving phage therapy. The results shown below are a continuation of the previous research. Both papers allow one to determine whether phage therapy induces production of neutralizing antiphage antibodies and whether they are associated with the results of the treatment.

## Materials and Methods

### Ethics Approval Statement

Experimental phage therapy was approved by the Bioethics Committee at the Wrocław Medical University (approval number KB-349/2005 with further amendments) and was conducted in accordance with the Declaration of Helsinki and national rules governing experimental therapy. Each patient gave informed consent prior to beginning the treatment. The study was approved by the same bioethical commission (approval number KB-414/2014).

### Patients Subjected to Phage Therapy and Healthy Volunteers

Adult patients with various infections (e.g., bone infections, sinus infections) resistant to antibiotic treatment received phage treatment under the therapeutic protocol entitled “Experimental phage therapy of drug-resistant bacterial infections, including MRSA infections” (Międzybrodzki et al., 2012). Patients (*n* = 20) treated in the Phage Therapy Unit in Wrocław, Poland with the *S. aureus* MS-1 phage cocktail were examined. Nineteen of them suffered from infections caused by methicillin-sensitive *Staphylococcus aureus* strains (MSSA); from one patient the *Staphylococcus lugdunensis* strain was isolated. All patients received treatment over the years 2012-2015. Specific data regarding patients examined in this study are summarized in **Table 1**. Sera of 10 healthy blood donors came from the Blood Donation Center, Clinical Military Hospital in Wrocław, Poland.

**Table 1:** List of examined patients from the Phage Therapy Unit in Wrocław, Poland.

Patient	Type of infection	Preparation used in phage therapy	Route of administration
1	Left shank infection	OP MS-1	Locally
2	Right hip joint fistula	MS-1	Locally
3	Chronic infection of the paranasal sinuses and the maxillary sinus	MS-1	Locally and orally
4	Ulceration of perianal area after colon resection	MS-1, since 77th day of therapy *S. aureus* A5/80 phage, after 5-month interruption *S. aureus* φ200 phage	All phages were applied locally and orally
5	Inflammation of the left tibia	MS-1	First locally, after 8 weeks of the treatment orally
6	Infection of the subcutaneous tissue with a thigh fistula	MS-1, since 105th day of therapy *S. aureus* 676/F phage	all phages were applied orally
7	Left elbow infection with active fistula	MS-1	Locally
8	Periprosthetic infection of the left hip	OP MS-1 TOP, since 42nd day of therapy *S. aureus* φ200 phage	OP MS-1 TOP phage cocktail was applied locally, φ200 phage orally
9	Inflammation of the left tibia	MS-1	Locally
10	Fracture of the ankle with ulceration	MS-1, after 8-month interruption *S. aureus* 676/F phage	Locally, after 8.5 months of the treatment orally, 676/F phage was applied only orally
11	Chronic infection of the paranasal sinuses and the throat	MS-1	locally and orally
12	Inflammation of the right calcaneus	MS-1	Locally
13	Right foot infection	MS-1	Locally
14	Chronic infection of the paranasal sinuses	MS-1, since 35th day of therapy *S. aureus* P4 phage	Orally, P4 phage was applied locally and orally
15	Chronic conjunctivitis, chronic sinusitis	MS-1, after two months treatment *S. aureus* A3/R phage	all phages were applied Locally and orally
16	Periprosthetic infection of the right hip	MS-1	Locally
17	Inflammation of the right femur	MS-1	Locally
18	Surgical wound infection of the belly with fistula	MS-1	Orally
19	Right thigh infection	MS-1	Locally
20	Chronic purulent inflammation of the right tibia	MS-1	Locally

The effectiveness of phage treatment was evaluated according to the scale from A to G (Międzybrodzki et al., 2012) where categories A-C were considered as good responses to the phage therapy (A – pathogen eradication, B – good clinical results, C – clinical improvement) and categories D-G were considered as inadequate responses to phage treatment (D – questionable clinical improvement, E – transient clinical improvement, F – no response to the treatment, G – clinical deterioration).

The details of patient treatment including routes of administration in different types of infections have been described earlier (Międzybrodzki et al., 2012).

### Phage Preparations

#### Testing Bacterial Susceptibility to Phages

The MS-1 phage cocktail consists of three lytic *S. aureus* phages – 676/Z, A5/80 and P4/6409. Among 458 tested MSSA strains of *S. aureus*, 73.4% were susceptible to the MS-1 phage cocktail. Effectiveness was lower for MRSA strains (from 28 tested strains 53.6% of them were susceptible to MS-1 phage cocktail). Detailed analysis of susceptibility to the MS-1 phage cocktail is presented in **Table 2**. The phage components of MS-1 cocktail were chosen to achieve optimal (maximal) lytic range based on the theoretical statistical analysis of the results of the sensitivity of staphylococcal strains against individual staphylococcal phages from our collection. The results shown in **Table 2** are presented for new collection of staphylococcal strains isolated from patients after introduction of MS-1 phage cocktail. Therefore, they represent a real staphylococcal sensitivity to MS-1 as well as to its individual phages. We are working on new, more efficient composition of staphylococcal phage cocktail based on the results and experience obtained from using MS-1. All our patients are treated with targeted phage preparations, which means that in each case we use only phages or phage cocktail which are lytic against a pathogenic bacterial strain isolated from the patient. Therefore, in all our MRSA cases treated with MS-1, the bacteria were susceptible to the phage cocktail. Otherwise, our MRSA patients were treated with another single staphylococcal phage from our collection. If we were not able to identify an active phage, patients were not qualified to experimental phage treatment.

**Table 2:** Susceptibility of *S. aureus* MSSA and MRSA strains to MS-1 phage cocktail (Weber-Dąbrowska et al., 2012; unpublished data).

	MS-1	676/Z	A5/80	P4/6409
Susceptible (MSSA)	336 (73.4%)	260 (72.8%)	252 (70.6%)	192 (53.8%)
Resistant (MSSA)	122 (26.6%)	97 (27.2%)	105 (29,4%)	165 (46.2%)
TOTAL	458 (100%)	357 (100%)	357 (100%)	357 (100%)
Susceptible (MRSA)	15 (53,6%)	4 (57.1%)	3 (42.9%)	2 (28.6%)
Resistant (MRSA)	13 (46,4%)	3 (42.9%)	4 (57.1%)	5 (71.4%)
TOTAL	28 (100%)	7 (100%)	7 (100%)	7 (100%)

Bacterial susceptibility to specific polyvalent staphylococcal phages from our collection was evaluated by spotting method (Ślopek et al., 1983; Chirakadze et al., 2009). The bacteria cultures were prepared in liquid broth medium. Next, the suspensions were spread on plates with solid agar medium. Plates were dried in incubator (37°C, 1.5 h). Drop of each phage lysate (10^7^-10^9^ pfu/ml of initial suspension) was spotted on plate surface, then plates were incubated (37°C, 6 h) and stored at 4°C until the following day. A positive result was recognized (under the therapeutic point of view) when confluent or semi-confluent lysis was observed.

#### Phage Propagation

All phages used in this study were propagated in liquid broth (LB). To each of five flasks, containing 160 ml of medium, 5 ml overnight host bacterial culture was added, mixed together and incubated for 1 h at 37°C. At the same time one large flask (3-5 l capacity) was filled with a mixture of LB (160 ml), 5 ml of high titre phage stock (at least 10^8^-10^9^ pfu/ml) and 5 ml of overnight host strain culture. After an 1 h incubation period the content of one of the five flasks was added to a large flask. This step was repeated every 30 min until the last flask was emptied. From the moment of the phage addition the culture was incubated for 10 h at 37°C. After this step the large flask was stored under refrigeration at 4°C. The following day, phage culture was filtered aseptically using membrane filters (0.22 μm) in a laminar flow cabinet and its titer was determined.

#### Therapeutic Phage Preparations

All examined patients (with two exceptions) received phage cocktail lysates (MS-1) produced by IBSS BIOMED S.A. in Kraków, Poland. According to the leaflet, the therapeutic phage dose was at least 5 × 10^5^ pfu/ml. One patient received a purified phage cocktail (OP MS-1) with a phage dose of at least 1 × 10^9^ pfu/ml of each phage suspended in phosphate buffered saline (PBS) with 10% addition of saccharose. Finally, one patient received an analogous purified phage cocktail deprived of saccharose (OP MS-1 TOP). Saccharose is used to maintain the stability of storage conditions, including structure preservation and specific interactions (Chang et al., 2005). Typically, phages were applied orally and/or locally 2-3 times a day in the amount of 5-10 ml per dose (each case was individually evaluated by a physician). Before each oral phage administration 10 ml of oral suspension of dihydroxyaluminium sodium carbonate (68 mg/ml) was applied as well.

#### Purified Phage Preparations

Due to high sensitivity of the ELISA technique, all phage preparations used in our examinations (676/Z, A5/80, and P4/6409) were purified according to a previously described method (Łusiak-Szelachowska et al., 2014) with some changes. After phage propagation in LB liquid medium, phage lysates were concentrated using Vivaflow 200 (tangential flow module) with Hydrosart membrane (the molecular weight cut-off is 30 000). The next step was removal of lysate contaminants from the phage suspension by size exclusion chromatography using a Sepharose CL-4B column (GE Life Sciences 26/100) under the following conditions: elution buffer 0.068 M phosphate buffer, pH 7.2; flow rate 2.2 ml/min; detection UV at 280 nm. Sepharose CL-4B is a cross-linked form of Sepharose, which is chemically and physically more resistant than Sepharose itself and offers better flow characteristics with the same selectivity. Purified phage particles were dialysed to PBS. The final titers ranged from 1.3 × 10^10^ to 3.5 × 10^10^ pfu/ml. To evaluate the level of endotoxins, the Endpoint Chromogenic Limulus Amebocyte Lysate (LAL) test was performed, according to the manufacturer’s instructions (Lonza). The LAL test is a quantitative test mainly for Gram-negative bacterial endotoxins. However, it is helpful to detect endotoxins in every phage preparation. As our findigs revealed, LPS can be present even in sterile peptone water. It happens because we use reusable glass tubes and LPS is well known for its very strong adhesion properties. Even relatively low dose of LPS at the beginning of phage propagation and purification is multiplied during the concentration process. The final levels of detected endotoxins in phage preparations varied from 994 to 1727 EU/ml, which gave no more than 20 EU/ml after dilution of phage preparations for ELISA purposes. Similar low values were detected in staphylococcal phage preparations used in phage therapy.

### Preparation of Serum Samples

All blood samples were taken before and during treatment, in some cases also after phage therapy. The sera were separated from heparinized blood samples by centrifugation at 1,500 *g* for 10 min and stored in 1-1.5 ml aliquots in a freezer at -70°C.

### Phage Inactivation

The phage neutralization by human sera was estimated as the rate of phage inactivation (*K* rate) by the method described in our previous work (Łusiak-Szelachowska et al., 2014). Fifty microliter of phage lysate (1 × 10^6^ pfu/ml) was added to 450 μl of diluted serum (from 1:10 up to 1:1500). Next, sample was incubated at 37°C for 30 min. After incubation the mixture was diluted 100 times with cold broth and the phage titer was determined. *K* rate was estimated using the equation:

(1) K=2.3×(D/T)×log⁡(P0/Pt)

where, *K* is the rate of phage inactivation, *D* is the reciprocal of the serum dilution, *T* stands for the time in minutes during which the reaction occurred (30 min. in this case), P0 is the phage titer at the start of reaction and Pt is the phage titer after reaction. A *K* rate less than 5 was considered as weak phage neutralization, between 5 and 18 as a medium level, and above 18 as a high level of phage neutralization.

### ELISA Procedure

Immune analysis was based on detection of the level of specific antiphage antibodies in human sera reacting with phage antigens using an indirect ELISA technique. We used three different types of secondary antibody to detect specifically human IgG, IgA, and IgM. In contrary to our previous results based only on raw absorbance values (Łusiak-Szelachowska et al., 2014), herein we established standard reference serum and final results have been provided as antibody units (AU). The immunological response was measured to each phage separately (*S. aureus* 676/Z, A5/80, and P4/6409 phages) as well as to the entire *S. aureus* MS-1 phage cocktail. Purified *S. aureus* phage preparations as antigens were diluted immediately before use in 0.05 M coating buffer (carbonate-bicarbonate buffer; Sigma-Aldrich) to obtain the titer at 5 × 10^8^ pfu/ml. The phage cocktail was prepared after diluting phages in coating buffer by mixing equal volumes of three different *S. aureus* phage solutions (676/Z, A5/80, and P4/6409).

At the beginning of the procedure, 96-well flat-bottom microplates (Nunc MaxiSorp; Thermo Scientific) were loaded with 100 μl of purified phage preparations as antigen-containing samples. Next, covered microplates were incubated for 1 h at 37°C and washed six times using PBS with 0.05% addition of Tween 20 (Sigma-Aldrich), which is a synthetic detergent helpful in removing non-binding residue antigens. Multiple washing was a mandatory stage in the procedure after each incubation period at 37°C. The washing step was performed by an automatic 96-needle microplate washer (HydroSpeed; Tecan) with a wash rate at 350 μl/s. The next step was to apply to microplate wells 200 μl of blocking protein (1% solution of casein sodium salt from bovine milk in PBS; Sigma-Aldrich) for blocking any non-specific binding sites. Microplates were then again incubated for 1 h at 37°C. As primary antibodies human serum samples, diluted in blocking solution with 0.05% addition of Tween 20 in the proportions 1:1000 and 1:10 000, were used. Each serum sample was applied in duplicate by pipetting 100 μl of the solution per well. After that, microplates were incubated for 1 h at 37°C. Specific antiphage antibodies bound with antigen were detected by secondary antibodies. We used three different secondary antibodies produced in goat linked with the enzyme horseradish peroxidase (HRP) binding human IgG, IgA or IgM (Sigma-Aldrich). All anti-human secondary antibodies were diluted in blocking solution with 0.05% addition of Tween 20 in the proportion 1:15 000 to obtain working concentrations at ∼0.5 μg/ml. Microplates were loaded with 100 μl/well of secondary antibody solution and again incubated for 1 h at 37°C. The final step was to apply 200 μl/well of a chemical substrate (*o*-Phenylenediamine; Sigma-Aldrich) suspended in 0.05 M phosphate-citrate buffer containing 0.03% sodium perborate (Sigma-Aldrich) that is converted by the enzyme into a color measured spectrophotometrically to determine the presence and quantity of antiphage antibodies. Microplates with a substrate were incubated at room temperature for 30 min in the dark. After the incubation period, microplates were read on a multiwell plate reader (Sunrise; Tecan) using Magellan 7.1 software at 450 nm with shaking immediately before the reading. One series of the whole procedure closed at 1 day. We avoided night storage of loaded microplates in refrigerated conditions as low temperatures had an influence on the repeatability of the results. The value of absorbance in blank wells (filled only with buffer) was subtracted from the tested samples.

To verify the reliability of the tests, a series of control samples was carried out on each microplate to detect possible errors. To evaluate the blocking properties of casein solution as well as to detect any non-specific binding of human antiphage antibodies (IgG, IgA, IgM) on microplate surface some wells were loaded with coating buffer (without phages as antigens). To detect unusual cross-reactivity between the antigen and the secondary antibody, some microplate wells contained a control human serum sample deficient in IgG, IgA and IgM (Sigma-Aldrich). Control serum was prepared according to the manufacturer’s instructions in the same solution as all human serum samples. Some wells were loaded only with secondary antibody and substrate to detect any unusual endogenous reaction product. Of note, none of the above-mentioned control samples ever gave higher values of absorbance than blank wells. This means excellent selection of procedure parameters and reagents (data not shown). No cross-reactivity was observed with any of the potential cross-reactants tested.

Establishing a reference standard serum encountered some difficulties. Contrary to previous studies (Miura et al., 2008; Dąbrowska et al., 2014) we worked only on a human model. We could not immunize healthy humans with phages for obvious reasons. No commercial kit for detection of the human antiphage antibodies is yet available on the market as well. Obtaining a sufficient amount of highly reactive patient’s serum was limited due to a small volume of blood samples taken from patients and a relatively weak signal obtained in the ELISA test. From several dozen samples we chose two representatives; one was used for assessing IgG and IgM antibody levels, and the second one was used for assessing IgA levels. Dilutions of standard sera were made in twofold steps starting with 1:100 for IgA and IgM and 1:1000 for IgG to generate a four-point standard curve for each type of antibody. The AU were then calculated from their absorbance values at 450 nm using the parameters estimated from each standard curve (Miura et al., 2008). The average value of each duplicate (per sample) was calculated. The dilution giving an optical density at 450 nm of ≤1 was assigned as 1 000 AU (AUs). Achieving high values of absorbance (∼1 at 450 nm) for IgG caused no problems for us. However, due to weak reactivity of serum samples, we were able to obtain maximum values of absorbance at 0.6 for IgM and 0.25 for IgA, respectively. Reference sera as standards were run on each tested microplate.

### Statistical Evaluation of the Data

The evaluation of statistical significance of differences between groups in the neutralization test was performed using Wilcoxon’s test (for dependent variables) and the Mann–Whitney *U* test (for independent variables).

Results carried out by ELISA technique were analyzed using Wilcoxon’s test (for dependent variables). For independent variables, we used the Mann–Whitney’s *U* test and Student’s *t*-test. To choose the appropriate statistical test we confirmed whether in groups of IgG, IgA, and IgM antibodies distributions were normal and whether there were homogeneous variances. In case of normal distribution and homogeneous variances we applied parametric Student’s *t*-test. In remaining cases we used nonparametric Mann-Whitney’s *U* test.

Significance was set at *p* < 0.05 and *p* < 0.001. Analysis was performed using the Statistica 10 software package.

## Results

As mentioned before, each patient subjected to experimental phage therapy was treated individually by a physician in charge according to the protocol of the experimental phage therapy employed at the Phage Therapy Unit. Depending on the overall health, the type of infection and its severity, a specific treatment was applied after determination of the sensitivity of isolated bacterial strains to the specific phage. Follow-up visits (during which blood samples were taken) took place at various time intervals in every single case. The number of follow-up visits varied from 1 to 11 depending on the course of the therapy. Sometimes the therapy was terminated by a physician (if eradication of infection was achieved or poor toleration occurred), while in other cases patients terminated therapy themselves due to the lack of expected improvement during treatment. Furthermore, the duration of the treatment varied from one month to over a year (with interruptions). The main criterion was patients’ clinical condition confirmed by laboratory data. It should be emphasized that it is not unusual for patients with chronic bacterial infections (in contrast to acute infection episodes) to be treated with antibacterial agents for months (Leekha et al., 2011). Thus, it is hard to summarize and standardize all collected data. Therefore, it was impossible in this experimental therapy to standardize sample collections similarly to protocols of clinical trials. Here, we decided to focus on serum samples obtained before therapy and on samples giving maximum levels of antiphage antibodies during the whole treatment (in the case of a single follow-up visit, we evaluated the one serum sample we had). These results were compared with the level of phage inactivation (*K* rate) measured in the neutralization test and with the overall outcome of phage treatment for each patient individually.

### Effect of Phage Treatment on the Level of Antiphage Antibodies

**FIGURE 1:**
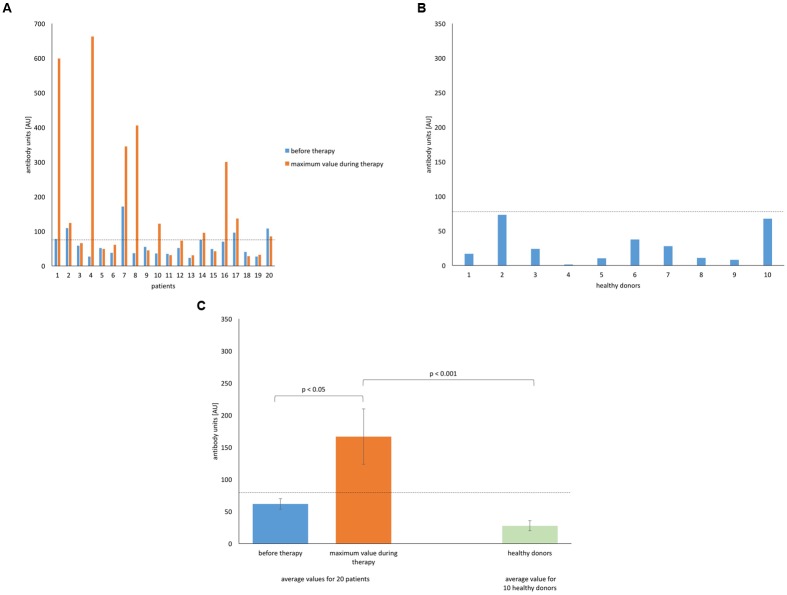
**Levels of IgG anti-MS-1 phage antibodies in human sera. (A)** Patients (*n* = 20) treated with *S. aureus* MS-1 phage cocktail. **(B)** Healthy (*n* = 10) donors. **(C)** Average values for patients treated with *S. aureus* MS-1 phage cocktail and healthy donors. Average values are plotted with the standard error (SE) indicated by the error bars. Median value for the group of patients during treatment is marked on each graph by a horizontal dotted line.

We focused mainly on the immune response to the *S. aureus* MS-1 phage cocktail as phage preparations were applied exactly in such composition to all patients. Here, we present the level of anti-MS-1 phage IgG (AU) measured by the ELISA test for all 20 examined patients (**Figure 1A**). Values obtained individually for all 10 healthy donors who had never been subjected to phage therapy are shown in **Figure 1B**. The comparison of these two groups (mean values) are presented in **Figure 1C**. Due to the high diversity in the obtained results, the median values for the group of patients during treatment were evaluated (**Figure 1A**). The median level of IgG antibodies reached the highest values in comparison to the levels of IgA and IgM and was 78.95 AU. None of the healthy volunteers reached this limit. The mean value of the level of antiphage antibodies (IgG) for the group of patients before treatment was 61.71 AU and 166.62 AU during treatment. For healthy donors the mean value was 27.75 AU. Earlier bacterial infections and exposure to relevant phages were probably responsible for higher initial antibody values in patients compared with healthy individuals.

**FIGURE 2:**
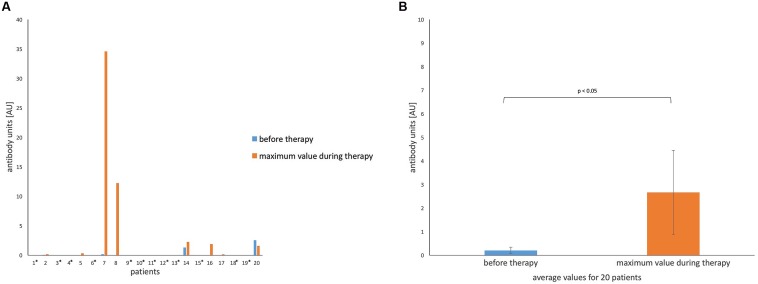
**Levels of IgA anti-MS-1 phage antibodies in human sera. (A)** Patients (*n* = 20) treated with *S. aureus* MS-1 phage cocktail. An asterisk next to the number of a patient means undetectable levels of antibodies before and during treatment. **(B)** Average values for patients treated with *S. aureus* MS-1 phage cocktail before and during treatment. Average values are plotted with the SE indicated by the error bars. The average value for 10 healthy donors was zero. Median value for the group of patients during treatment was zero as well.

In contrast to the results described above, levels of antiphage IgA were the lowest in the group of patients as well as in the group of healthy donors (**Figures 2A,B**) in comparison to the levels of IgG and IgM. In fact, we did not detect any antiphage IgA in sera of healthy people. Similar results were obtained in patients (60% of examined patients indicated undetectable levels of IgA before and during the whole treatment). This phenomenon occurred even in patients with sinus infections, where the mucosal immune system was stimulated by local administration of phages. Possibly, higher levels of IgA would be detected in saliva instead of serum. The median value of IgA obtained for the group of patients during treatment was zero as well. The mean value of the level of antiphage antibodies (IgA) for the group of patients before treatment was 0.2 AU and 2.66 AU during treatment.

**FIGURE 3:**
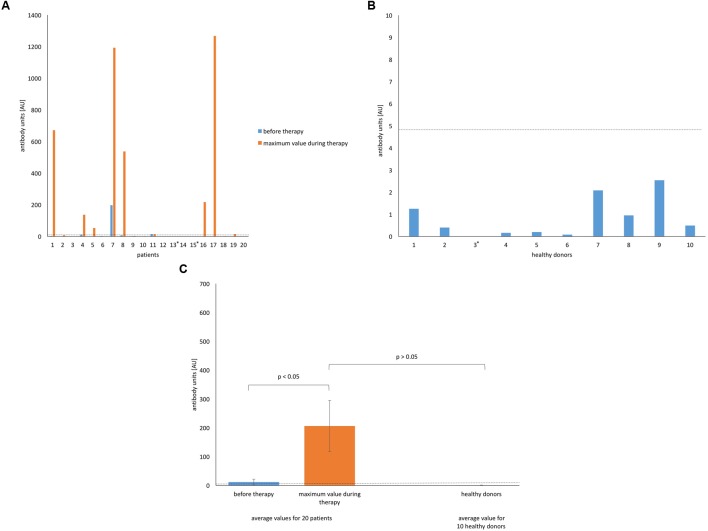
**The levels of IgM anti-MS-1 phage antibodies in human sera. (A)** Patients (*n* = 20) treated with *S. aureus* MS-1 phage cocktail. **(B)** Healthy (*n* = 10) donors. An asterisk next to the number of a healthy donor means an undetectable level of antiphage antibodies. **(C)** Average values for patients treated with *S. aureus* MS-1 phage cocktail and healthy donors. Average values are plotted with the SE indicated by the error bars. Median value for the group of patients during treatment is marked on each graph by a horizontal dotted line.

The levels of anti-MS-1 phage IgM were the most surprising, as we observed an astonishingly high spread between the lowest and highest values in some patients (**Figure 3A**). Samples of two patients reached levels over 1 000 AU as a response to local phage administration (patients 7 and 17). In another two cases we did not detect any IgM elicited by phage before or during phage therapy (patients 13 and 15). Despite high levels of IgM in some cases, the median value in the group of patients during treatment was only 4.99 AU. The group of healthy subjects was characterized by relatively low levels of IgM antiphage antibodies, which were close to zero (**Figures 3B,C**). The mean value of the level of antiphage antibodies (IgM) for the group of patients before treatment was 11.84 AU and 205.89 AU during treatment. For healthy donors the mean value was 0.81 AU.

Three patients (patients 7, 8, and 16 in **Figures 1**–**3**) with markedly higher levels of all antibody isotypes (IgG, IgA, IgM) were treated by phages locally. However, in cases with low levels of IgG and IgM antiphage antibodies and with undetectable levels of IgA antiphage antibodies (numbers 13 and 19 in **Figures 1**–**3**) phages were applied likewise locally. These results are consistent with our earlier report, where no relationship between the route of administration and ELISA results was observed (Łusiak-Szelachowska et al., 2014). Statistical analysis of ELISA results in patients and healthy donors are summarized in **Tables 3** and **4**.

**Table 3:** Statistical analysis of ELISA results in 20 patients (response to MS-1 phage cocktail).

Type of antibody	Mean level of antiphage antibodies (AU) before therapy ± SE	Mean level of antiphage antibodies (AU) during therapy ± SE	Statistical significance (*p*)
IgG	61.71 ± 8.19	166.62 ± 43.06	0.008^∗^
IgA	0.2 ± 0.14	2.66 ± 1.79	0.049^∗^
IgM	11.84 ± 9.8	205.89 ± 88.71	0.002^∗^

*^∗^Wilcoxon’s test*.

**Table 4:** Statistical analysis of ELISA results in 20 patients compared to 10 healthy donors (response to MS-1 phage cocktail).

Type of antibody	Mean level of antiphage antibodies (AU) in healthy people ± SE	Mean level of antiphage antibodies (AU) in patients during therapy ± SE	Statistical significance (*p*)
IgG	27.75 ± 7.86	166.62 ± 43.06	<0.001^∧^
IgA	0	2.66 ± 1.79	0.305^#^
IgM	0.81 ± 0.28	205.89 ± 88.71	0.067^∧^

*^∧^Mann-Whitney’s U test; ^#^Student’s t-test.*

**FIGURE 4:**
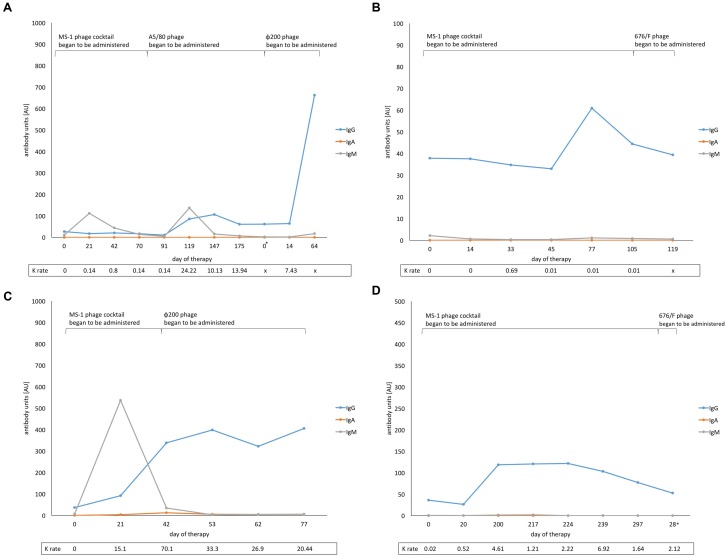
**Levels of anti-MS-1 phage antibodies (IgG, IgA, IgM) before and during phage therapy and antiphage activity of sera (AAS, *K* rate) in four patients with the largest number of control visits. (A)** Patient 4; ^∗^means 5-month break between successive cycles of phage treatment, **(B)** Patient 6, **(C)** Patient 8, **(D)** Patient 10; ^∗^means 8-month break between successive cycles of phage treatment. *x*, *K* rate was not estimated.

A long-term study of the four patients with the largest numbers of control visits showed meaningful differences in the levels of particular types of antiphage antibodies (**Figures 4A–D**). Apparently, IgA antibodies in sera were not involved in the humoral immune response during treatment as even long-term exposure (several months) to phage antigens did not induce secretion of IgA. In the case of patient 4 (**Figure 4A**), the *S. aureus* MS-1 phage cocktail was applied only for the first 76 days of therapy (*S. aureus* A5/80 and *S. aureus* φ200 phages were applied afterward). However, even a change of medication resulted in a noticeably stronger immune response by IgG antibodies to the MS-1 phage cocktail over a year after its administration. No changes in secretion of IgA antibodies were observed. In accordance with expectations, a marked increase of IgM was noted before the IgG boost directly after initiation of the treatment and after change of the phage preparation (from *S. aureus* MS-1 phage cocktail to *S. aureus* A5/80 phage). Analogously, even more rapid growth of IgM levels at the beginning of the treatment was noted in patient 8 (**Figure 4C**). Such dependence was not observed in another two cases (**Figures 4B,D**). IgG antibodies were mostly responsible for the humoral immune response in patients undergoing phage treatment. They persisted in the blood for several months during phage administration. All four patients analyzed in **Figure 4** received, at a certain stage of the treatment, another staphylococcal phage (different from the MS-1 phage cocktail). We did not evaluate the immune response to other phages used in treatment due to a lack of purified specific phage preparation.

### Immunogenicity Depending on the Type of Phage

**FIGURE 5:**
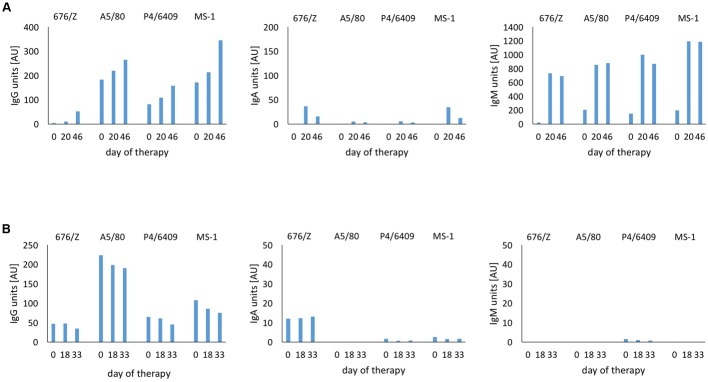
**Immunogenicity depending on the type of phage in patients treated with *S. aureus* MS-1 phage cocktail. (A)** Patient 7. **(B)** Patient 20. The immunological response to each phage from the MS-1 phage cocktail was examined in the same way as phage cocktail. Purified single *S. aureus* phage preparations as antigens were diluted immediately before use to obtain the titer at 5 × 10^8^ pfu/ml. The phage cocktail was prepared after diluting single phages in coating buffer by mixing equal volumes of three different *S. aureus* phage solutions (676/Z, A5/80, and P4/6409).

As stated above, beside investigations on the MS-1 phage cocktail, we measured likewise the immunological response to each phage from the cocktail separately. We chose two patients with a similar course of treatment. One of them showed a relatively high level of antiphage antibodies (especially IgA and IgM), while the second one revealed a much lower immune response. We noted that the response rate may depend not only on the type of applied phage but also on the type of antibody involved in binding to phage antigen. For IgG antiphage antibodies the most immunogenic (excluding the MS-1 phage cocktail) was *S. aureus* A5/80 phage, while for IgA it was *S. aureus* 676/Z phage (even if considering generally low values for IgA antiphage antibodies). We did not observe such coincidence for IgM antiphage antibodies. In patient with high levels of IgM antibodies the response to each phage reached a value over 500 AU after 20 days of the treatment. In the second case, practically no phage-induced production of IgM antibodies was observed (**Figures 5A,B**). Based on these results, we can assume that differences in the phages’ immunogenicity are related to the various structure of phage proteins, which is consistent with previous reports describing animal models (Capparelli et al., 2007; Dąbrowska et al., 2014; Majewska et al., 2015).

### Correlation between the Levels of Antiphage Antibodies and Antiphage Activity of Human Sera

Stimulation of neutralizing antibodies has been one of the arguments of opponents of phage therapy. We observed a correlation between increased level of antiphage antibodies detected in the ELISA procedure and higher rate of phage inactivation (*K*) mostly for IgG and IgM antibodies. No correlation was observed for IgA antibodies. Determining which type of antibody (IgG or IgM) is characterized by the strongest neutralizing properties toward phages is difficult, as the obtained results for IgG and IgM coincide with each other in almost every case with *K* > 18. However, it should be mentioned that even a very high rate of phage inactivation (*K* > 18) does not necessarily mean an unfavorable outcome of phage treatment. All changes in the levels of antiphage antibodies (IgG, IgA, IgM) measured by the ELISA test in patients before and during treatment were statistically significant (**Table 3**) at *p* < 0.05. Changes in the rate of phage inactivation (*K*) observed before and during phage therapy were statistically significant as well (**Table 5**) at *p* < 0.001. Results regarding mean *K* rate in healthy donors compared to patients during treatment are presented in **Table 6**. Summary data related to the levels of antiphage antibodies, rate of phage inactivation and clinical results of phage therapy are presented in **Table 7**.

**Table 5:** Statistical analysis of results from neutralization test in 20 patients (response to MS-1 phage cocktail).

Type of phage used in phage therapy	Mean *K* rate before therapy ±SE	Mean *K* rate in patients during therapy ±SE	Statistical significance (*p*)
*S. aureus* MS-1 phage cocktail	0.02 ± 0.01	23.78 ± 12.13	<0.001^∗^

*^∗^Wilcoxon’s test.*

**Table 6:** Statistical analysis of results from neutralization test in 20 patients compared to 10 healthy donors (response to MS-1 phage cocktail).

Type of phage used in phage therapy	Mean *K* rate in healthy subjects ±SE	Mean *K* rate in patients during therapy ±SE	Statistical significance (*p*)
*S. aureus* MS-1 phage cocktail	0.003 ± 0.002	23.78 ± 12.13	<0.001^∧^

*^∧^Mann-Whitney’s U test.*

**Table 7:** Immune response and clinical outcome of phage treatment in 20 examined patients.

Patient	IgG (AU) before therapy	IgG (AU) during therapy^∗^	IgA (AU) before therapy	IgA (AU) during therapy^∗^	IgM (AU) before therapy	IgM (AU) during therapy^∗^	Phage inactivation (*K*) before therapy	Phage inactivation (*K*) during therapy^∗^	Clinical outcome of phage therapy
1	77.82	598.84	0	0	2.07	671.4	0	204.49	F
2	108.91	124.04	0.06	0.17	0.21	6.56	0.01	0.14	F
3	58.44	65.74	0	0	0.07	0.09	0.02	0.13	E
4	26.8	662.56	0	0	10.99	136.68	0	13,94	E
5	51.72	48.87	0	0.34	0	52.82	0.03	10.84	F
6	37.82	60.92	0	0	2.15	1.12	0	0.69	E
7	171.45	345.1	0.19	34.59	197.35	1193.08	0.02	140.85	B
8	36.47	405.44	0	12.26	6.87	536.94	0	70.1	A
9	54.9	45.01	0	0	1.23	3.43	0	0.128	B
10	36	121.74	0	0	0.55	1.79	0.02	6.92	A
11	34.87	30.94	0	0	14.36	13.15	0.02	0.4	D
12	51.66	72.78	0	0	0.07	0	0	0.11	B
13	23.09	30.59	0	0	0	0	0.12	0.4	F
14	74.96	95.51	1.31	2.27	0	0.41	0.01	0.95	E
15	48.65	42.17	0	0	0	0	0.01	0.09	C, F^#^
16	69.92	300.28	0	1.9	0	216.57	0	10.26	D
17	95.96	136.56	0	0.16	0.61	1267.52	0.04	0.52	C
18	40.25	28.01	0	0	0.08	1.74	0.02	0.11	F
19	26.74	32.1	0	0	0	14.53	0.01	14.58	E
20	107.82	85.13	2.55	1.59	0.14	0.06	0	0.01	F

*^∗^Maximum values achieved during treatment; ^#^C, chronic sinusitis; F, chronic conjunctivitis.*

### Rates of Inflammatory Markers in Patients Treated with Phages

We also analyzed our data in the context of the selected inflammatory markers (data not shown) in patients before and during treatment. The inflammatory markers in patients’ blood were assessed by commercial laboratory methods. Two classical inflammatory markers, CRP (C-reactive protein) and ESR (erythrocyte sedimentation rate) were considered. They are believed to be the most valuable markers for evaluating a patient’s clinical status with respect to inflammation, infection, trauma or malignant disease (Saadeh, 1998; Międzybrodzki et al., 2009; Assasi et al., 2015). In the majority of cases both markers remained constant (with slight variations within the values) or were even lower during treatment than before phage therapy. In some cases, both markers decreased despite constantly increasing levels of antibodies (IgG and IgM) during treatment. Such data are partially consistent with previous results. Międzybrodzki et al. (2009) indicated a statistically significant reduction in mean CRP concentrations in patients during the first five weeks of phage treatment. Jończyk-Matysiak et al. (2015) observed that phage therapy had no effect on the level of inflammatory markers, ESR and CRP, when all data from patients observation were analyzed. Data are only partially consistent because the different patient cohorts, with various infections and undergoing different course of the treatment have been evaluated. It should be pointed out that purified phage preparations were applied in two cases (patient 1 was taking OP MS-1, whereas patient 8 was taking the OP MS-1 TOP phage preparation). Surprisingly, these two patients receiving purified phage preparations showed increased values of CRP during treatment (from 5.71 before therapy to 17.39 mg/l after two months of continuous treatment in patient 1 and from 5.9 to 8.52 mg/l after five weeks of treatment in patient 8). Furthermore, the purified phage preparations had no effect on lower induction of antibodies at all. In fact, patients treated with OP MS-1 and OP MS-1 TOP preparations had the highest levels of IgG and IgM antibodies measured by the ELISA technique within the whole tested group.

## Discussion

The results from earlier reports are consistent with our current examinations and clearly show that bacteriophage can induce production of antiphage antibodies (mostly IgG and IgM) which may be responsible for phage inactivation. However, the immune response to phages depends on many different factors (duration of the treatment, phage dosage and route of administration). The immune status of a patient is no less significant. Previous reports indicated that several patients from our Phage Therapy Unit had immune deficits caused by infections, antibiotic treatment, etc. (Kurzępa-Skaradzińska et al., 2014). Górski et al. (2006) assumed that phage translocation in patients may be much higher than in healthy people as the gut barrier in disease is often much more permeable to microorganisms. Furthermore, in patients with immunodeficiencies, applied phages are believed to have longer viability (Borysowski and Górski, 2008). Some sources have noted that toxins produced by pathogenic bacteria in humans may have an inhibitory effect on their immune response (Gobert et al., 2007). Hentzer et al. (2001) found that temperate *P. aeruginosa* phages contribute to production of modified biofilm by pathogenic *Pseudomonas* strains, which is more resistant to antimicrobial treatments and activity of the immune system cells. As stated before, such data are still limited, especially when relating to human models. Pescovitz et al. (2011) noted that φX174 phage circulated for 3 to 4 days after intravenous application in healthy people until IgM antibodies completely inactivated phage particles before day 7. IgG antibodies were induced as well. In investigations by Bruttin and Brussow (2005), no humoral immune response was observed after oral administration. The authors suggested that no substantial amount of T4 phage ever appeared in their blood. Our group obtained similar results (Łusiak-Szelachowska et al., 2014). Although, we did not demonstrate the presence of phages in blood, such suggestion seems to be reasonable. Oral application induced the weakest immunological response, but, in this study, similar low levels of antibodies were observed in many patients after local administration as well. In our study we were unable to assess the direct relationship between the route of administration and the intensity of the immune response, as the majority of the tested group received phages locally or both locally and orally (only in two cases were phages applied entirely orally).

Some sources indicate that antiphage antibodies may be present in patients’ sera before phage treatment or even in sera of completely healthy people. Kucharewicz-Krukowska and Ślopek (1987) observed the presence of specific antiphage antibodies in 23% of patients before phage treatment. Our earlier work (Łusiak-Szelachowska et al., 2014) indicated that a low level of antiphage activity of patients’ sera could be detected before treatment. Dąbrowska et al. (2014) obtained congruous findings when investigating the level of anti-T4 phage IgG in the human population. Over 80% of healthy individuals were found to have antiphage antibodies in their sera. Those antibodies, so-called “natural antibodies” (Górski et al., 2012), may be a result of high prevalence of phages, which are well known for their vast abundance in almost every environment, even in the water supply system of European cities (Weber-Dąbrowska et al., 2014), and can induce antibody production in healthy controls. Our trials showed the presence of antiphage IgG antibodies in the group of 10 healthy donors at similar levels as in the group of patients before implementation of phage therapy. We also found correlations between the levels of antiphage IgA (undetectable levels in the group of healthy people and exceptionally low values in patients before and at the time of the treatment). Differences were found in the levels of antiphage IgM. A few patients exhibited relatively high levels of antiphage IgM antibodies before phage application. Those levels increased rapidly at the beginning of the treatment.

The results obtained for two patients administered purified phage preparations provide interesting knowledge for further development of phage therapy. Generally, the patients subjected to treatment in the Phage Therapy Unit in Wrocław, Poland receive phage lysates (phage particles suspended in liquid bacterial medium) instead of purified preparations. Phage lysates may contain macromolecules derived from the host bacteria and culture medium. Their presence raises an important concern regarding the safety of the therapy (Szermer-Olearnik and Boratyński, 2015). Our preliminary results suggest that those concerns are unfounded. Based on the above observations, we can presume that application of phage lysates does not cause any deleterious effects compared to purified phage preparations. However, emphasis should be laid on phage titer in both phage preparations, which was four ranges lower in phage lysates, making them possibly less immunogenic. It appears that phage dose, not the level of purification of phage solution, plays the most important role in immunogenicity of therapeutic phage preparations in humans. However, larger cohorts of patients are needed to draw definite conclusions.

Data from animal models are more numerous. Results obtained by Stashak et al. (1970) and Sulakvelidze and Barrow (2005) indicated the appearance of antiphage antibodies in animals which are able to inactivate phages during treatment. The latest reports have presented similar results. In a murine model, specific immunization to T4 head proteins decreased phage activity *in vitro* and *in vivo* in a group pre-immunized with phage Hoc protein but only *in vitro* in a group pre-immunized with gp23 protein (Dąbrowska et al., 2014). Differences in the immunogenicity of phage structural proteins were reported by other authors. Capparelli et al. (2007) described two staphylococcal phages, wild type and its mutant driven by the mice immune system, which were serologically distinct. The mutant was persistent in neutralizing antibodies in the mouse circulation, whereas the wild phage was almost completely inactivated within 2 days after intravenous application. A long-term study of antibody induction in mice by T4 phage applied orally in very high doses (4 × 10^9^ pfu/ml thus making approximately 2 × 10^10^ pfu/mouse daily; mice were fed with T4 phage in drinking water for 100 days) showed a significant increase in antibody levels (IgG in sera after 36 days of treatment and IgA in feces after two months of continuous treatment). The increased IgA level antagonized gut transit of active phage (Majewska et al., 2015).

Finding similarities among animal and human models encounters difficulties due to certain limitations of this study. No doubt, animal experiments can be planned more accurately. We were not able to assess phage viability in human tissues as was done in animal investigations (Hodyra-Stefaniak et al., 2015) or in the gut. The results gained so far from human stool samples (Łusiak-Szelachowska et al., 2008) did not reflect the full scope of events taking place within the gut during phage treatment. Similar obstacles were faced by Sarker et al. (2016). Considering the immunogenicity of phages in the animal gut, the term “rather low” has been used (Majewska et al., 2015). However, one cannot directly extrapolate those studies in mice to the clinic.

## Conclusion

Overall, the majority of studies to date show that phages may induce a humoral immune response in humans. Interpretations regarding the strength of this response are often contradictory or unclear. As described above, patients who showed the highest level of antiphage antibodies and the highest antiphage activity of their sera ended phage treatment with good clinical results or even with full recovery. The significance of these findings is not to be underestimated, as the results from animal experiments cannot be simply transferred to a human model. Our studies confirm and extend our earlier work indicating that phage therapy may induce various levels of antibody formation which does not necessarily affect the outcome of therapy. Evidently, further studies are needed to shed more light on phage-dependent immune responses and their significance for the success or failure of therapy.

## Author Contributions

MŻ, MŁ-S, EJ-M, BW-D, BO, AK, AG: designed the experiments, analyzed the data, wrote and revised the manuscript, approved the version to be submitted. RM, WF, PR: collected and analyzed data from patients, revised the manuscript, approved the version to be submitted. RM, WF, PR, AG: conducted phage treatment in patients. MŻ, MŁ-S, BO, AK: performed the experiments.

## Conflict of Interest Statement

The authors declare that the research was conducted in the absence of any commercial or financial relationships that could be construed as a potential conflict of interest.
